# Candida albicans: a cause or a consequence of esophageal intramural pseudo-diverticulosis

**DOI:** 10.11604/pamj.2019.33.280.19601

**Published:** 2019-08-02

**Authors:** Imen Akkari, Elhem Ben Jazia, Soumaya Mrabet, Imen Jemni

**Affiliations:** 1 Gastroenterology Department, Farhat Hached Hospital, Sousse, Tunisia

**Keywords:** Esophageal intramural pseudo-diverticulosis, antifungal treatment, candida albicans

## Abstract

Esophageal intramural pseudo-diverticulosis is a rare disease of unknown etiology. It is characterized by multiple pseudodiverticula with segmental or diffuse involvement of the esophagus. We report, the case of a 78-year-old male who suffered from severe dysphagia. Diagnosis of esophageal intramural pseudo-diverticulosis was based on endoscopic and radiologic explorations. Histological analysis of esophageal mucosal biopsies has shown the presence of candida albicans. Antifungal treatment leads to spectacular improvement of dysphasia. Subsequently, the patient presented a cardio-respiratory failure and died despite adequate treatment.

## Introduction

Esophageal intramural pseudo-diverticulosis (EIPD) is a rare condition of unknown etiology described in 1960 by Mendel [1]. It is characterized in radiologic explorations by the presence of multiples small flask shaped out pouching in the wall of esophagus [2-4] and it can be associated to severe stricture [3]. The treatment is based on medical treatment especially IPP and dilatation in the case of severe stricture [2, 3, 5]. We report the case of a 78 male patient who suffered from a severe dysphagia. He was diagnosed with an EIPD after the endoscopic end radiologic explorations. The presence of candida albicans was objectified in esophageal biopsy; therefore, an antifungal treatment was administrated with clinical improvement. Later the patient developed a cardio-respiratory failure and died despite an adequate treatment.

## Patient and observation

**Figure 1:**
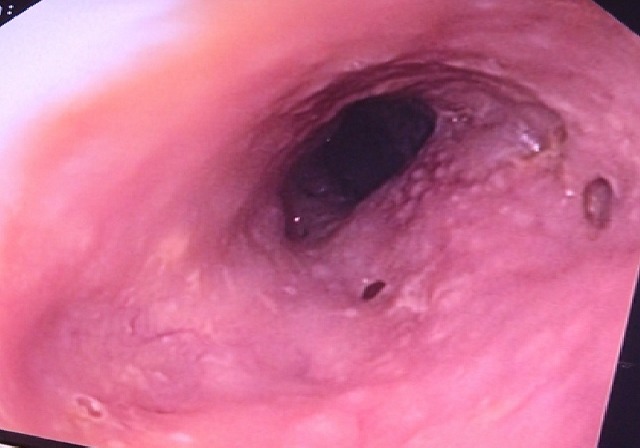
Esophageal intramural pseudo-diverticulosis revealed by upper endoscopy

A 78-year-old man without any medical history presented with dysphagia. He suffered from weight loss for one year estimated at 30 kg, but he didn't consult any hospital until a continuous and mixed dysphagia appeared 2 weeks ago. The patient was a heavy smoker with a history of 78 packs per year and a former heavy drinker who stopped alcohol consumption for 10 years. The physical examination was normal but the laboratory data revealed an iron deficiency anemia (hemoglobin level at 8.1g/dL), a low albumin level (22g/L) and a biological inflammatory syndrome with an increase of gammaglobuline level (23.87g/L) and an accelerated sedimentation rate: 120 at the first hour. Oesogastroduodenal fibroscopy revealed an innumerable tiny opening of pseudodiverticula (Figure 1) with the presence of a white and creamy liquid emerging from the orifices. The esophageal lumen was slightly reduced on the third inferior part of oesophagus. The barium oesophagogram revealed an esophageal stricture on the low thoracic esophagus; on the oral side of the narrowing mucosal, there was a mucosal irregularity with tiny collections of barium outside the wall of the esophagus. The computed tomography revealed a hypertrophied esophageal wall with small gas collection corresponded to pseudodiverticula. The biopsies showed a chronic inflammation and detected the presence of candida albicans. The patient has been treated with antifungal medicine (fluconazol for ten days) with spectacular improvement of digestive symptoms. One month later, the patient presented to the emergency with cardio-respiratory failure. He died despite an adequate treatment.

## Discussion

The EIPD is a rare entity first described on 1960 by Mandal [1] that may occur at any age (1-86 year) with masculine predominance (sex ratio: 1,4) [2]. It corresponds to a dilatation of secretary ducts of the submucosal glands [5]. The most common symptom observed in 80% of patients is dysphagia. But it can be sometimes asymptomatic [6]. Radiologic examinations showed a specific sign in the form of small bottle, flask-shaped protrusions of the mucous membrane [5]. In the present case, collections were greater as occurs in fistula formation; this might be probably due to a bridging between adjacent diverticula. On endoscopy, this dilatation appears as a small opening or depression in the esophageal wall that are found on only 25% of patients [6]. An esophageal stricture is observed in 70-90% of patients and is most common in the upper esophagus [6]. In this present case, pseudodiverticula were diffusely present through the entire esophagus with a slight reduce of the lower esophagus lumen. The etio-pathogeny of this disease still unclear until now. It could be congenital due to a mechanic disorder [2]. Or acquired due to gastroesophageal reflux, esophageal stenosis, inflammatory diseases of the esophagus (including candidiasis) [2, 5]. Some cases of EIPD with a concomitant eosinophilic oesophagitis has been described [7, 8], a possible relationship between these two disorders has been suggested. In these cases [8], pseudodiverticula was mainly observed in mild to distal esophagus, patient was younger, have a higher risk of food obstruction and have an atopy predisposition. Esophageal candidiasis has been also described in the pathogeny of EIPD. It is observed in approximately 50% of patients, but it could be related to stasis [2]. Takashi [3] reported, in 2012, a case of an advanced stage of esophageal stricture derived from EIPD in which antifungal treatment was effective leading to long lasting resolution of dysphagia, in this case, *Candida albicans* was a cause of exacerbation of a preexisting EIPD by the concomitant inflammatory cellular infiltration of the esophageal wall [3]. In the present case, a creamy liquid was present in the first endoscopic exploration. This esophageal candidiasis could be the cause of the EIPD or a consequence of a prolonged EIPD. Regardless of the esophageal candidiasis involvement, the research of this infection is important in order to treat and obtain a clinical improvement. The later deterioration of the patient might be due probably to a complication of a persistent EIPD after the initial clinical improvement. This cardio-respiratory failure was noted also in a case reported by Szczesna [5] despite adequate treatment. Long terms observations show no regression of EIPD despite appropriate treatment and improvements in the general conditions [5, 9, 10].

## Conclusion

This case report reveals the therapeutic difficulties on the management of EIPD and the importance of the systematic search for esophageal candidiasis in order to obtain at least a clinical amelioration.

## Competing interests

The authors declare no competing interests.
